# Comparative analysis of the remineralization potential of CPP–ACP with Fluoride, Tri-Calcium Phosphate and Nano Hydroxyapatite using SEM/EDX – An *in vitro* study

**DOI:** 10.4317/jced.55941

**Published:** 2019-12-01

**Authors:** Charisma Thimmaiah, Priya Shetty, Sowmya B. Shetty, Srikant Natarajan, Nithya-Annie Thomas

**Affiliations:** 1 MDS, Assistant Professor, Department of Pedodontics and Preventive Dentistry, Manipal College of Dental Sciences, Mangalore. Manipal Academy of Higher Education, Manipal, Karnataka India; 2 MDS, Reader, Department of Pedodontics and Preventive Dentistry, AJ Institute of Dental Sciences, Kuntikana, Mangalore Karnataka, India. Rajiv Gandhi University of Health sciences; 3 MDS, Professor and Head, Department of Pedodontics and Preventive Dentistry, AJ Institute of Dental Sciences, Kuntikana, Mangalore Karnataka, India. Rajiv Gandhi University of Health sciences; 4 MDS, Professor and Head, Department of Oral Pathology and Microbiology. Manipal College of Dental Sciences, Mangalore. Manipal Academy of Higher Education, Manipal, Karnataka, India; 5 MDS, Senior Lecturer, Department of Pedodontics and Preventive Dentistry, Indira Gandhi institute of dental sciences, Nellikuzhi, Kothamanagalam, Kerala -India. Kerala University of health sciences

## Abstract

**Background:**

In recent years, the non-invasive management of non cavitated caries lesions using remineralization systems to repair the enamel have received more attention from the scientific community. Aim: To quantitatively evaluate the remineralization potential of Casein phosphopeptide-amorphous calcium phosphate-fluoride(CPP-ACPF), Tri-calcium phosphate(TCP) & Nano-hydroxyapatite(nHAP) using Scanning Electron Microscopy(SEM) and Energy dispersive X-ray Analysis(EDX).

**Material and Methods:**

40 enamel specimens were prepared, and immersed in demineralising solution at a pH of 4.4 for 96 hours at 37°C, to induce artificial carious lesions. Remineralization was carried out for a period of 30 days using CPP-ACPF, TCP, nHAP. The specimens were evaluated for calcium and phosphorus content using SEM-EDX.

**Results:**

The Ca/P mass % after remineralization was significantly higher with CPP-ACP-F and TCP-F followed by nHAP.

**Conclusions:**

CPP-ACP-F and TCP can promote significant remineralization of incipient carious lesions. These are excellent delivery vehicles available in a slow release amorphous form to localize calcium, phosphate and fluoride at the tooth surface.

** Key words:**Remineralization, in vitro; CPP-ACP fluoride, Nano-hydroxyapatite, Tri-calcium phosphate, SEM/ EDX.

## Introduction

The current concept of caries process is based on accumulation of numerous episodes of demineralization and remineralization, rather than a unidirectional demineralization process (1). In an incipient stage, prior to cavitation, when there is loss of minerals from the dental hard tissues demineralization takes place. However, the repair of a lesion can occur when the calcium and phosphate gradients are reversed and they diffuse inwards rather than outwards this process is termed as remineralization (2).

The non-invasive treatment of early carious lesions by remineralization has the potential to be a major advance in clinical management of dental caries (3). Products containing calcium, phosphate and fluoride in their bio available forms have claimed to enhance remineralization over products containing only fluoride (4).

 A calcium based system was prepared by reacting beta-Tri-calcium phosphate (β-TCP) with Sodium Lauryl Sulfate (SLS) using a mechanochemical ball milling process to form a functionalized calcium phosphate (5).Tri-calcium phosphate has been included in a tooth crème with 0.2 % sodium fluoride marketed as Clinpro™ Tooth Crème.

The use of milk and milk products, having a protective effect against the development of dental caries, has been a novel concept in remineralization. The anticariogenic properties of milk is due to the presence of casein, calcium and phosphate, which are responsible for resistance to acid dissolution (5,6). One such, remineralizing agent which has been extensively studied and accepted is casein phosphopeptide amorphous calcium phosphate (CPP-ACP). Fluoride incorporated into CPP-ACP is shown to have a higher remineralization potential (4). GC Tooth Mousse Plus® is a water based cream containing CPP-ACP with 0.2% Sodium fluoride (CPP-ACPF).The advantage is the availability of calcium, phosphate and fluoride in one product.

With the recent advances in nanotechnology, the size of particles has decreased to usually 0.1 to 100nm and some modifications in their shape, yielding highly bioactive calcium, phosphate compounds have higher potential for penetration into the porosities of the demineralized area with a potential of remineralization (7,8).Aclaim® is a Nano hydroxyapatite toothpaste with 1% concentration of Nano hydroxyapatite. Nanohydroxyapatite crystals penetrate the enamel pores and act as a template in the precipitation process, promoting crystal integrity and growth.

Scanning Electron Microscopy has been a useful tool in dentistry for research, it allows the visualization of images at high magnification (50X-10.000X and above). It is used in conjunction with Energy dispersive X-ray analysis, a micro analytical technique to quantitatively estimate the amount of calcium phosphate ratio (9).

## Material and Methods

This was an *in vitro* experimental study, which was conducted in A.J. Institute of Medical Sciences, Mangalore in collaboration with Manipal Institute of Technology, Innovation Center, Manipal.

-Ethical Clearance 

Approval for the research project was granted by Institutional Ethics Committee, A.J. Institute of Dental Sciences, Mangalore, India. An informed consent was obtained before the onset of the study.

-Sample size calculation

**Figure 1:**
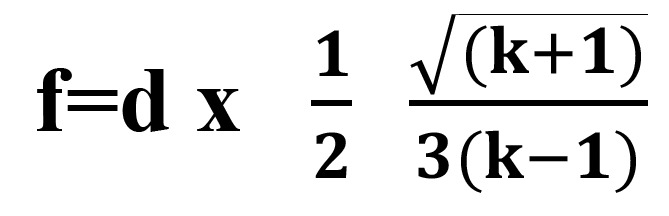
Formula.

The sample size was calculated using the formula, (Fig. 1):

where k= number of groups, d is calculated using the difference between the highest and the lowest means. It was calculated by using data from an article by Lata S, Varghese NO, Varughese JM (10).

A total of 40 enamel specimens were needed. This calculation was based on the assumption that the confidence interval is 95%; power 80%, level of significance was set at 0.001 a true difference between treatments, adjusted to 4 units. This was based on the calculations by SPSS software version 20.

**Figure 2:**
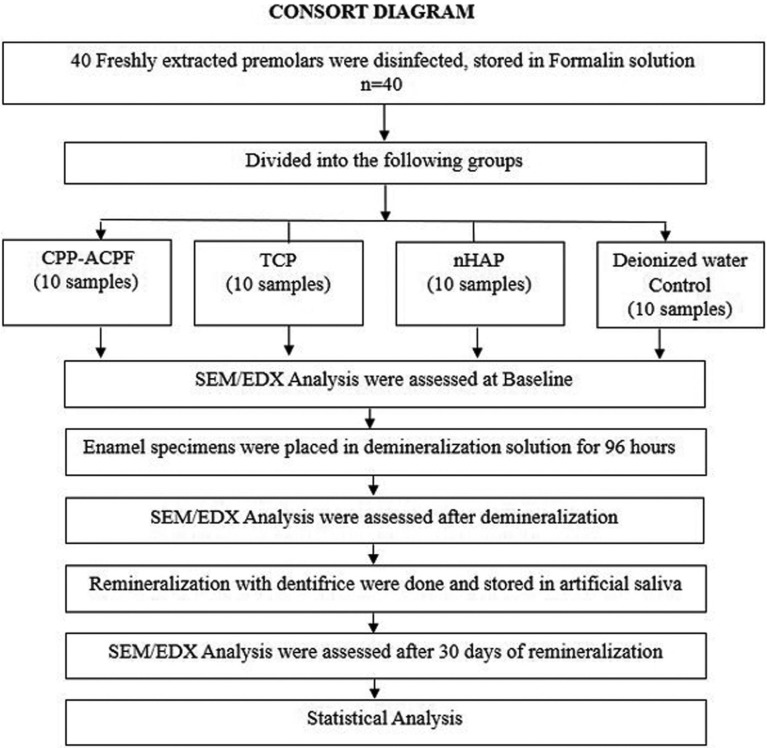
Consort diagram depicting the study protocol.

-Sample Preparation:

In order to avoid errors regarding the reliability and reproducibility, a pilot study was done prior the original study with 12 samples evaluating the remineralization potential with SEM/EDX analysis. The enamel specimens were evaluated by a single examiner who was trained in SEM/EDX Analysis (Fig. 2).

Forty enamel specimens were prepared from buccal surfaces of caries free extracted human premolar teeth. Teeth with fracture of either crown or root, caries lesion including white spot lesion, hypoplastic lesions detected visually, teeth with developmental defects or any other crown deformities, teeth with any restoration were excluded from the study.

Firstly, the soft tissue debris were cleaned using an ultrasonic scaler and inspected for cracks, hypoplasia and white spot lesions, to be excluded. Teeth were then sectioned horizontally using a diamond disc with micro motor straight hand piece (NSK Japan) at 15,000 rpm at the level of CEJ (Cemento Enamel Junction), separating the crown part of the tooth. The cusp and occlusal surface of the crown was then removed following the same technique.

Following sample preparation windows were created of dimension 4mm×4mm×2mm in size, and the rest of the area on the sample was completely resistant to acid attack by carefully coating nail varnish exhibiting a narrow rectangular area on the enamel surface. Elemental (Ca/P) analysis was done at baseline and structural analysis was done to observe the surface morphology of the enamel using SEM-EDX. Subsequently, the teeth were immersed in the demineralizing solution (5 mL/specimen) which was prepared using 2mmol/L Calcium (Calcium Chloride - CaCl2), 2mmol/L Phosphorus (Monopotassium phosphate - K2HPO4), 0.075mmol/L acetate at a pH of 4.3 for 96 hours at 37°C to produce artificial enamel carious lesions (9,10).Structural analysis was then performed using SEM-EDX analysis for the estimation of loss of mineral (Calcium and Phosphorus) after demineralization for each specimen.

40 sectioned enamel specimens were randomly assigned to 4 treatment groups of 10 sections each and subjected to the following treatment protocols (Fig. 2).

GROUP 1- GC Tooth Mousse Plus® (Recaldent GC Asia Dental)

GROUP 2- Clinpro™ Tooth crème (3M™ ESPE)

GROUP 3- Aclaim® toothpaste (Group pharmaceuticals Ltd)

GROUP 4-Artificial Saliva

The teeth in each group were treated daily with their respective remineralizing agents by using a cotton applicator tip, after which the specimens were washed with deionized water and placed in artificial saliva at a temperature of 37°C, in an incubator mimicking the oral temperature. Artificial saliva was prepared using 3.90mM Trisodium Phosphate(Na3PO4), 4.29mM Sodium Chloride(NaCl),17.98mM Potassium Chloride(KCl), 1.10mM Calcium Chloride (CaCl2),0.08mM Magnesium Chloride (MgCl2) ,0.50mM Sulfuric acid (H2SO4) ,3.27mM Sodium Bicarbonate (NaHCO3) and distilled water (10).The artificial saliva was changed every 24hrs. The control was artificial saliva because this substance represents natural saliva, which does not demineralize enamel. It was predicted that tooth enamel would demineralize when treated with artificial saliva. After 30 days of remineralization, the samples from each group were subjected to SEM- EDX analysis.

-Specimen Preparation for SEM- EDX

The enamel specimens were subjected to sputtering using a thin film of silver foil as a sputter (11). After sputtering, the specimens were subjected to evaluation of mineral content (mass/atomic percentage) using Energy dispersive X-ray analysis (EDX).The digital outputs of the EDX values were interpreted numerically as Ca/P ratios at baseline, after demineralization and remineralization.

## Results

Comparison of Ca/P mass % values after remineralization by using one way ANOVA test showed that the mean value of CPP-ACPF (1.913) was highest. This difference is statistically significant with p value <0.001( Table 1).

**Table 1:**
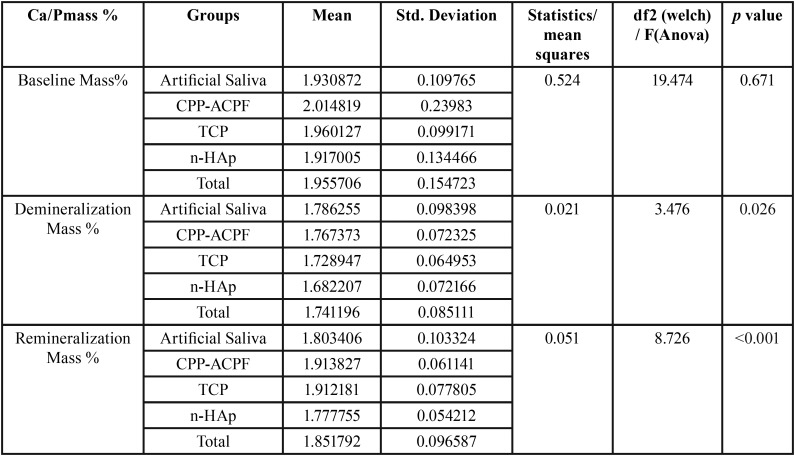
One way analysis of variance test with post hoc Tukey test.

**Figure 3:**
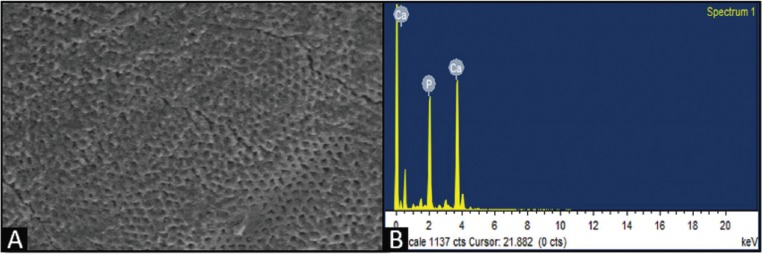
A) SEM image post demineralization of CPP-ACPF. B) EDX interpretation post demineralization of CPP-ACPF.

The SEM images after 96 hours of demineralization showed numerous micro porosities with less smooth surfaces of enamel and loss of aprismatic enamel, suggestive of demineralization (Fig. 3A).

The digital outputs of the EDX values were interpreted numerically as Ca/P ratios at various intervals at baseline, post demineralization and 30 days post remineralization. There was a decrease in the Ca/P mass percentage post demineralization when compared to baseline (Fig. 3B).

**Figure 4:**
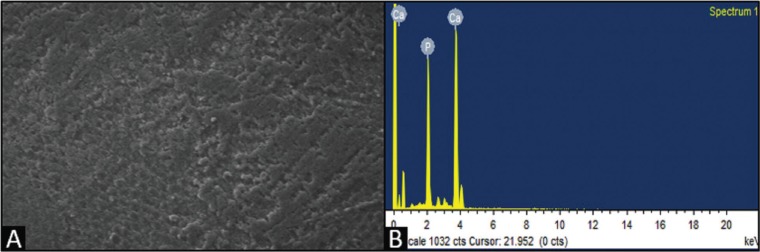
A) SEM image post remineralization of CPP-ACPF. B) EDX interpretation post remineralization of CPP-ACPF.

SEM images of remineralized surface shows less evidence of micro porosities. Amorphous deposits were seen when the specimens were remineralized. Greater deposits were observed in CPP-ACPF group quantitatively (Fig. 4A) .There was an increase in the Ca/P mass percentage post remineralization when compared to demineralization (Fig. 4B).

Post Hoc Analysis was done for each group at three different intervals which showed that the comparison of difference between mean values of Ca/P mass % after demineralization and remineralization was highest in TCP with Fluoride (0.183).This difference was statistically significant with a *P* Value <0.001 ( Table 2).

**Table 2:**
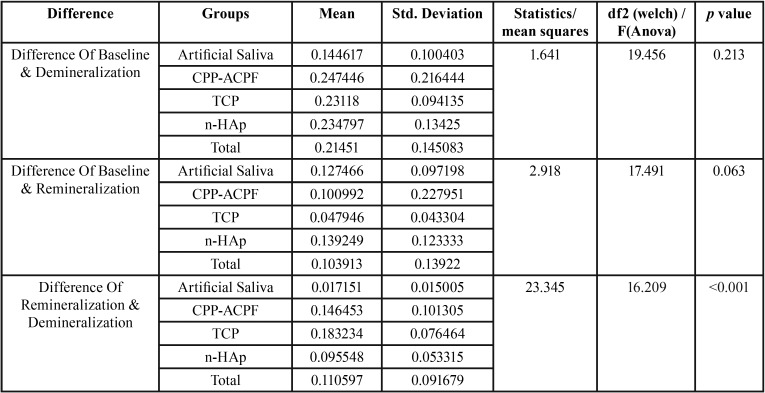
Inter group comparison assessing the difference at various intervals.

Paired t test was used for intra group comparison after demineralization and remineralization. On Comparison the mean values of Ca/P mass % after remineralization was higher and the value was statistically significant for all individual groups ( Table 3).

**Table 3:**
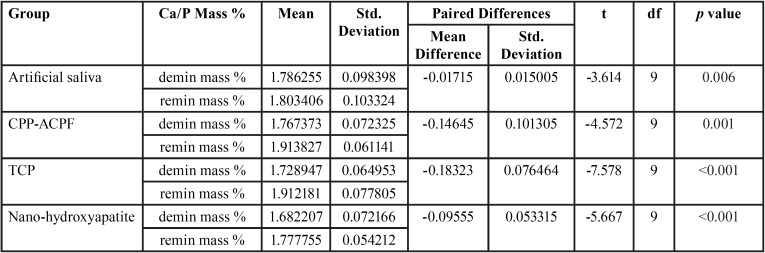
Intra group comparison for Artificial saliva, CPP-ACPF, TCP, and Nano-hydroxyapatite after demineralization (demin) and remineralization (remin).

## Discussion

The modulation of the demineralization-remineralization balance is the key to prevention of dental caries (2). Until recently the concept of conventional treatment for carious lesions involved caries excavation and replacement with a restorative material. However, with decades of research, the focus for the effective management of carious lesions is now shifted to the early detection of lesions and use of noninvasive techniques.Attempts to achieve this reversal state have been successfully made by using dentifrices. The non-invasive treatment of early caries lesions by remineralization is a major advantage in clinical management and remineralization bridges the traditional gap between prevention and surgical procedures (12). Central to this vision is the ability to detect caries lesions at an early stage and correctly quantify the degree of mineral loss, ensuring that the correct intervention is instituted.

The synergetic anticariogenic effect of CPP-ACPF and TCP has the added benefit of providing calcium and phosphate in the surrounding medium when there is an acid challenge. These are excellent delivery vehicles available in a slow release amorphous form to localize calcium, phosphate and fluoride at the tooth surface (13). Nano hydroxyapatite has a potential of remineralizing the tooth structure. It is hydrophilic and has greater surface area than the conventional hydroxyapatite crystals. Hence, they have better wettability and form a thin but strong layer on enamel surface that bonds to tooth structure (14).

The pH cycling model has been used widely to investigate caries-preventive agents on the dynamics of enamel demineralization and remineralization (Featherstone, 1996, Ten Cate *et al.*, 2006) (15). In the present study, pH cycling was implemented for a period of 30 days in order to allow sufficient time to produce changes in the demineralized enamel specimen. This model involved exposing the enamel specimens for 96 hours of demineralization using Acetic acid buffer at a pH of 4.4.

Remineralization with tri calcium phosphate with Fluoride showed increase in Ca/P mass percentage after demineralization similar to a study done by Namritha Patil *et al.* (16) who reported that TCP + fluoride based products perform better than CPP-ACP based products in remineralizing artificial enamel caries and Incorporation of fluoride into CPP-ACP compounds enhanced the remineralizing capacity.

Jayarajan J *et al.* (4) showed that the added benefit of fluoride (NaF 0.2%) to CPP-ACPF (Tooth Mousse-Plus) showed marginally more amount of remineralization than CPP-ACP alone (Tooth Mousse). Tschoppe *et al.* (17) evaluated the effects of a toothpaste containing NHA on enamel and dentin remineralization and showed that NHA toothpaste had greater efficacy for enamel and dentin remineralization than amine fluoride toothpaste. Bajaj M. *et al.* (18) compared CPP-ACP, Tri-calcium phosphate and Hydroxyapatite on remineralization of artificial caries like lesions on primary enamel, showed that Hydroxyapatite had better remineralization efficacy when compared to CPP-ACP and Tri-calcium phosphate.

In the present study, EDX analysis showed that the percentage gain of Ca and P after remineralization was greatest with CPP-ACPF when compared to other agents. SEM evaluation showed that CPP-ACPF had less evidence of micro porosities and greater amorphous deposits followed by TCP and Nano-hydroxyapatite.

One possible limitation of the current study is that the lesions created in the study to replicate the demineralized surfaces might not represent white spot lesions completely. Remineralization *in-vitro* may be quite different when compared with dynamic complex biological system, which usually occurs in oral cavity *in vivo*. Thus, direct estimation to clinical conditions must be exercised with caution.

## Conclusions

CPP-ACP with Fluoride(GC Tooth Mousse Plus™) and Tricalcium Phosphate (Clinpro tooth crème™) showed better remineralization potential compared to nano-hydroxyapatite (Aclaim®). Hence topical application of CPP-ACP with Fluoride, Tricalcium Phosphate, Nano-hydroxyapatite containing toothpastes have a definite role in remineralization of initial carious lesions.
